# Health insurance and the distribution of healthcare use in Rwanda’s Vision Umurenge Programme: evidence from the Seventh Integrated Household Living Conditions Survey

**DOI:** 10.1080/16549716.2026.2641401

**Published:** 2026-03-10

**Authors:** Jean de Dieu Harerimana

**Affiliations:** a Department of Planning, Monitoring and Evaluation, Health Financing, Ministry of Health, Kigali, Rwanda; b Health System Strengthening, Health Financing, Clinton Health Access Initiative, Inc., Kigali, Rwanda

**Keywords:** Health insurance, healthcare utilization, equity, Rwanda, social protection

## Abstract

**Background:**

Rwanda’s community-based health insurance (CBHI) has achieved near-universal enrollment, yet inequities in healthcare use remain. Understanding whether coverage translates into equitable utilization is critical for advancing universal health coverage (UHC). This study aims to examine how social protection complements health insurance in promoting equitable healthcare access.

**Objective:**

To examine the relationship between insurance coverage and healthcare utilization in sectors targeted by the Vision Umurenge Programme (VUP), assess socioeconomic inequities, and evaluate the complementary role of social protection.

**Methods:**

This study analyzed cross-sectional data from 15,039 households in VUP sectors using the 2023–2024 Seventh Integrated Household Living Conditions Survey. Socioeconomic inequality was measured using Erreygers-corrected concentration indices and need-standardized horizontal inequity analysis. Insurance effects were estimated using survey weighted logistic regression, propensity score matching, and doubly robust inverse-probability-weighted regression adjustment.

**Results:**

Insurance coverage was 85.8%, yet only 25.5% reported any formal healthcare utilization in the 12 months preceding the survey, including outpatient visits, inpatient admissions and preventive services. Utilization showed stronger pro-rich concentration (E =  +0.066) than coverage (E =  +0.026) and need-standardized analysis confirmed residual inequity. Insurance increased utilization by +14.6% points in regression models and ~+12% points in causal estimators. Participation in VUP components, particularly direct support, was consistently associated with higher service use.

**Conclusions:**

In a high coverage setting, persistent pro-rich inequities highlight the role of non-financial barriers such as indirect costs and service readiness. Layering social protection with insurance and strengthening primary care delivery is critical to convert nominal coverage into equitable healthcare access.

## PAPER CONTEXT

- **Main findings:** In sectors covered by Rwanda’s Vision Umurenge Programme (VUP), health insurance significantly increases the likelihood of seeking formal care, but utilization remains disproportionately concentrated among wealthier households.
- **Added knowledge:** Even after adjusting for health need, pro-rich inequities in utilization persist, highlighting that barriers beyond premiums such as transport costs, lost income, and other indirect expenses limit access for the poorest.
- **Global health impact for policy and action:** Integrating health insurance with targeted social protection and investments in supply-side readiness can help reduce non-financial barriers, strengthen effective coverage, and promote more equitable progress toward universal health coverage.

## Background

Achieving Universal Health Coverage (UHC) remains one of the most pressing challenges across low- and middle-income countries (LMICs). While most LMICs have expanded public health insurance over the past two decades, progress has often been uneven: coverage rates remain low, voluntary enrollment leads to adverse selection, and financial protection is frequently incomplete [1]. Against this backdrop, Rwanda stands out as an exceptional case. Rwanda has expanded health insurance enrollment to more than 90% of the population, a level rarely seen in resource-constrained settings [2].

A cornerstone of this achievement was the rollout of *Mutuelle de Santé* (CBHI), which shifted the system away from reliance on full user fees toward a co-payment model. Historically, out-of-pocket (OOP) spending dominated and impeded access for the poor, with average annual household OOP of Rwf 16,883, and 11.9% of households faced catastrophic health spending, meaning healthcare costs exceeded a substantial share of income [3–5]. Building on rapid expansion that surpassed 85% by the early 2010’s, Rwanda were insured, reflecting both strong government commitment and broad public uptake.

The financing model deliberately combined tax-based subsidies with sliding-scale household premiums, ensuring sustainability while embedding progressive cross-subsidization [6]. Despite these achievements, national household surveys confirm that insurance expansion has coincided with rising utilization across socioeconomic groups. According to the seventh Integrated Household Living Conditions Survey (EICV7, 2023/24), 71% of individuals with a reported health issue in the 4 weeks prior to the survey had a medical consultation up from 57% in EICV5 (2016/17) indicating improved healthcare utilization among the insured population [7].

Beyond enrollment, growing evidence examines how insurance affects financial risk protection and service use among vulnerable populations in Rwanda. Studies document reductions in catastrophic spending for high-cost events (e.g. cesarean sections) but also highlight residual post-discharge and indirect costs that continue to burden poor households [8]. Expansion has coincided with greater utilization among rural and poorer groups, though inequalities have not been fully eliminated [9]. Despite this progress, approximately 10% of the population remains uninsured and uncovered, with the uptake barriers concentrated among the youngest household heads and among poorer wealth quintile [10]. Cross-country comparisons within sub-Saharan Africa further indicate substantial heterogeneity, with Rwanda performing relatively well on enrollment but still facing socioeconomic gradients in use [11].

These persistent gradients reflect a poverty – health feedback loop – a self-reinforcing cycle in which poor health reduces household income through lost productivity and medical expenditures, while poverty in turn limits access to preventive and curative care, thereby perpetuating poor health outcomes that insurance alone cannot break, thereby motivating the need for complementary social protection measures. The Vision Umurenge Programme (VUP), launched in 2008, represents a critical pillar in Rwanda’s strategy to reduce both financial and non-financial barriers to care. Through Direct Support (unconditional cash transfers), Classic Public Works, Expanded Public Works, and Nutrition-Sensitive Direct Support, VUP targets the most vulnerable households.

By easing liquidity constraints and facilitating insurance enrollment or renewal, the programme can offset transport and time costs and income losses associated with care seeking, thereby enhancing the ‘effective coverage’ of essential services. Evidence further suggests that social protection layered with insurance yields larger gains in service use and financial protection than either instrument alone [12]. In addition, recent experimental and review level evidence from sub-Saharan Africa supports these findings. A cluster-randomized controlled trial conducted in Zambia demonstrated that cash transfer programme’s significantly increased healthcare utilization among the poorest households [13]. Complementing this, a scoping review of cash transfer interventions across multiple settings reported consistent improvements in service uptake, although the evidence regarding broader health outcomes, including morbidity and mortality, remains mixed and inconclusive [14].

Despite extensive research on insurance expansion in Rwanda and other LMICs, three critical gaps remain. First, limited evidence exists on equity in healthcare utilization in near-universal coverage settings where financial barriers have been substantially reduced. Most equity analyses focus on coverage gaps rather than utilization inequities among the insured [15]. Second, insufficient integration of causal inference methods with equity-focused concentration analysis limits the ability to attribute observed inequities to insurance versus other factors [11,16]. Third, scarce empirical evidence examines how social protection mechanisms complement health insurance to address non-financial barriers such as transport costs, time burdens, and opportunity costs [17,18]. This study addresses these gaps by combining need-standardized equity metrics with rigorous causal estimation in Rwanda’s high-coverage context, specifically examining the complementary role of the Vision Umurenge Programme in promoting equitable healthcare access.

## Methods

### Study design, data, and sample frame

This study conducts a cross-sectional analysis using microdata on 15,039 households from the Seventh Integrated Household Living Conditions Survey (EICV7, 2023–2024). To align inference with the policy context, the analytic sample is restricted to administrative sectors participating in the Vision Umurenge Programme (VUP). All descriptive statistics and model-based estimates are therefore representative of households residing in these sectors. For transparency, the analysis reports the effective sample size and survey-weighted population totals.

#### Inclusion/exclusion

The analysis includes de *jure* households with complete responses on insurance status, healthcare utilization, wealth/consumption, and survey design variables (weights, strata, primary sampling units). Observations with *unknown/expired* insurance at interview were excluded from the insured group; item non-response was addressed.

#### Units and timing

The sampling unit is the household (*N* = 15,039), following EICV7’s multi-stage stratified design. However, the primary outcomes insurance coverage and healthcare utilization are measured at the individual level for all de jure household members. Descriptive statistics and regression analyses therefore report individual-level prevalence rates, while equity analyses account for household clustering through survey design weights and primary sampling units. This approach reflects standard practice in health equity research using household surveys, where individual health outcomes are nested within household sampling frames. Household-level characteristics (e.g. female-headed household, urban residence, wealth quintile) are assigned to all members within each household.

### Variables and measurement

#### Insurance

The primary exposure is health insurance coverage ($\mathrm{In}s_{i}\in \left\{0,1\right\}$) defined as valid coverage at interview. Three categories are distinguished: (i) community-based health insurance (*Mutuelle de Santé*); (ii) social insurance, and (iii) private plans. Subsidy status is proxied by *Ubudehe* categories. Coverage by scheme and subsidy is presented descriptively; robustness checks restrict exposure to CBHI only.

#### Utilization outcomes

The main outcome is any utilization of formal healthcare in the last 12 months ($\mathrm{Us}e_{i}\in \left\{0,1\right\}$). Secondary outcomes disaggregate into: (a) outpatient visit in the last 4 weeks; (b) any inpatient admission in the last 12 months; (c) preventive contact (e.g. vaccination, antenatal care), and (d) number of visits. For the intensive margin (number of visits; positive out-of-pocket amounts), two-part models are used.

#### Health need and access covariates

Need covariates include age groups, sex, disability, recent illness, chronic conditions, pregnancy in household, and under-five presence. Access covariates include education of household head, household size, an urban indicator, travel-time/distance proxies to the nearest facility (or sector level facility geocodes are available), and district fixed effects.

#### Financial protection

Out-of-pocket (OOP) spending is summarized annually. Values were winsorized at the 99th percentile. Positive OOP amounts were modeled with a generalized linear model (GLM; log link, Gamma family) after a binary part for any OOP.

### Survey design and estimation

The survey uses a multi-stage, stratified design. Design weights, strata and primary sampling units were declared using Stata 19.5 survey settings. Unless otherwise noted, point estimates and standard errors are design-based via linearization. For procedures not natively survey-aware (e.g. some matching diagnostics), the analysis (i) incorporated design weights in the first-stage estimation and (ii) report uncertainty using primary sampling units as clusters (bootstrap or cluster-robust standard errors).

#### Wealth ranking

Socioeconomic rank ($R_{i})$ was computed as the survey-weighted mid-point fractional rank in the distribution of (equivalized) consumption/wealth; ties were handled using weighted mid-ranks.

### Equity metrics

Concentration and Erreygers indices were computed following Wagstaff & van Doorslaer [19]. For an outcome $y_{i}$ with mean μ and range [a, b], the standard concentration index is:

$$C_{\mathrm{std}}=\frac{2}{\mu }\mathrm{Co}v_{\omega }\left(y_{i},R_{i}\right)$$

Where $\mathrm{Co}v_{\omega }$ denotes the survey-weighted covariance. For bounded outcomes, the Erreygers index is reported as

$$E=\frac{4\mu }{b-a}C_{\mathrm{std}}=\frac{8}{b-a}\mathrm{Co}v_{\omega }\left(y_{i},R_{i}\right)$$

and for binary outcomes $\left(a=1,b=1\right)$ this simplifies to $E=4\;\mu C_{\mathrm{std}}=8\;\mathrm{Co}v_{\omega }\left(y_{i},R_{i}\right)$

#### Need standardization and horizontal inequity

Need-expected utilization is estimated ${\hat{y}}_{i}^{\mathrm{need}}$ from a model including only need covariates (age, sex, disability, morbidity, pregnancy, under-5). Indirect standardization is implemented and define the need-standardized outcome: $y_{i}^{IS}=y_{i}-{\hat{y}}_{i}^{\mathrm{need}}+\overset{ˉ}{y}$. Horizontal inequity is then: $HI_{E}=E\left(y^{\mathrm{IS}}\right)=E\left(y\right)-E\left({\hat{y}}^{\mathrm{need}}\right)$. Equity metrics (concentration curves, Erreygers, need standardization, and decomposition) use design weights.

### Determinants of utilization

A survey-weighted logit model was estimated for any utilization:

$$\mathrm{Pr}\left(\mathrm{Us}e_{i}=1\right)=\mathrm{logi}t^{-1}(\beta_{0}+\beta_{1}\mathrm{In}s_{i}+\beta_{2}^{T}\mathrm{Nee}d_{i}+\beta_{3}^{T}\mathrm{Acces}s_{i}+\beta_{4}^{T}\mathrm{Wealt}h_{i}+\beta_{5}^{T}\mathrm{Pro}g_{i}+\gamma_{d}$$

where $\gamma_{d}$ are district fixed effects.

Average marginal effects (AMEs), area under the curve (AUC), calibration statistics, and predicted probabilities across wealth quintiles and programme components are reported. Continuous predictors are centered and, where skewed, log-transformed.

### Causal estimand and selection on observables

#### Target estimand

The analysis pre-specifies the Average Treatment Effect on the Treated (ATT) and the Average Treatment Effect (ATE). All causal analyses respect survey design where feasible and explicitly state the estimand.

#### Propensity scores and matching

Propensity scores $p=\mathrm{Pr}(\mathrm{In}s_{i}=1|X)$ are estimated by survey-weighted logit including all pre-treatment covariates: need, access, wealth, education, household size, and district fixed effects. Common support is documented with density/overlap plots and trim observations with extreme p. For ATT, nearest-neighbor matching with replacement is implemented and a caliper on the logit of the propensity score (0.2 SD) and kernel matching (Epanechnikov kernel, bandwidth chosen by cross-validation). Post-matching balance is assessed using standardized mean differences (target |SMD|<0.10), variance ratios, and Love plots. Uncertainty is obtained via Abadie–Imbens analytic variance with primary sampling units as clusters, benchmarked to cluster bootstrap.

#### Weighting estimators and doubly robust estimation

The inverse probability weights (ATE, ATT) were estimated and overlap weights targeting common support. The analysis also implemented inverse-probability-weighted regression adjustment (IPWRA), with separate outcome models by treatment status. Diagnostics include positivity (min/max p), effective sample size, and influence checks.

#### Heterogeneity

We pre-specify interactions between insurance status and (i) wealth quintiles and (ii) programme components in the logit model, and report AMEs by subgroup with design-based standard errors.

## Results

### Descriptive statistics

Table 1 summarizes household characteristics in programme sectors. Women represent 51.9% of the population (*N* = 32,204; SE = 0.19), and 28.6% of individuals live in urban areas (*N* = 17,776; SE = 0.46). The population age structure shows that 15.1% are aged 0–5 years (*N* = 9,393), 33.0% are aged 6–17 years (*N* = 20,486), 46.8% are aged 18–59 years (*N* = 29,068), and 5.1% are aged 60 years or older (*N* = 3,163). The mean household size is 6.0 members (*N* = 62,110; SE = 0.03).

**Table 1.** Household characteristics in programme sectors.

Characteristic	% or Mean	SE	N
**Demographic characteristics**			62,110
Female (%)	51.85	0.19	32,204
Urban residence (%)	28.62	0.46	17,776
**Age group (%)**			
0–5 years	15.13	0.15	9,393
6–17 years	32.98	0.21	20,486
18–59 years	46.80	0.21	29,068
≥60 years	5.10	0.11	3,163
**Household size (mean)**	5.98	0.03	62,110
**Socioeconomic status (wealth quintiles)**			
Quintile 1 (poorest)	22.39	0.58	13,906
Quintile 2	20.51	0.47	12,739
Quintile 3	19.36	0.46	12,024
Quintile 4	18.62	0.45	11,565
Quintile 5 (richest)	19.12	0.58	11,876
**Health need indicators**			
Illness in last 4 weeks (%)	25.14	0.29	15,608
Any disability (%)	8.79	0.15	5,459
**Health insurance coverage**			
Covered by any insurance (%)	85.57	0.014	54,098
CBHI (Mutuelle de Santé) (%)	92.11	0.34	49,830
Social insurance (RSSB/MMI/School) (%)	6.65	0.30	3,598
Private/NGO/Other (%)	1.23	0.15	665
**Household Head**			
Male-Headed	96.09	0.077	23,908
Female-Headed	3.91	0.077	973
**Healthcare utilization**			
**Outpatient consultation in last 4 weeks (%)**	98.09	0.15	14,045
**Vision Umurenge Programme (VUP) participation**			
Direct Support (DS) (%)	1.31	0.12	814
Classic Public Works (cPW) (%)	3.09	0.22	1,920
Expanded Public Works (ePW) (%)	1.95	0.16	1,211
Nutrition-Sensitive Direct Support (NSDS) (%)	4.10	0.21	2,546
Number of VUP components received (mean)	0.10	0.004	

Notes: Survey-weighted means with design-based standard errors (SE).

Households are distributed across wealth quintiles, with 22.4% in the poorest quintile (*N* = 13,906; SE = 0.58), 20.5% in the second quintile (*N* = 12,739; SE = 0.47), 19.4% in the third quintile (*N* = 12,024; SE = 0.46), 18.6% in the fourth quintile (*N* = 11,565; SE = 0.45), and 19.1% in the richest quintile (*N* = 11,876; SE = 0.58). Illness in the 4 weeks preceding the survey was reported by 25.1% of individuals (*N* = 15,608; SE = 0.29), and 8.8% reported any disability (*N* = 5,459; SE = 0.15).

Health insurance coverage was reported by 85.6% of individuals (*N* = 54,098), with 92.1% of insured individuals covered by Community-Based Health Insurance (*N* = 49,830; SE = 0.34), 6.7% by social insurance (*N* = 3,598; SE = 0.30), and 1.2% by private or NGO-based schemes (*N* = 665; SE = 0.15).

Male-headed households account for 96.1% of households (*N* = 23,908; SE = 0.08), and female-headed households account for 3.9% (*N* = 973; SE = 0.08). Among individuals reporting illness, 98.1% reported an outpatient consultation in the previous 4 weeks (*N* = 14,045; SE = 0.15). Participation in Vision Umurenge Programme components includes Direct Support (1.3%; *N* = 814; SE = 0.12), Classic Public Works (3.1%; *N* = 1,920; SE = 0.22), Expanded Public Works (2.0%; *N* = 1,211; SE = 0.16), and Nutrition-Sensitive Direct Support (4.1%; *N* = 2,546; SE = 0.21). The mean number of VUP components received is 0.10 (SE = 0.004).

### Equity in coverage and utilization

Table 2 presents Erreygers-corrected concentration indices. Insurance coverage shows a small but statistically significant pro-rich concentration with an index of +0.0255 (SE = 0.0019, *p* < 0.001). This indicates that coverage is only slightly higher among wealthier households. In contrast, healthcare utilization displays a stronger pro-rich concentration, with an index of +0.0657 (SE = 0.0079, *p* < 0.001). The concentration is nearly three times larger than that for coverage, and the difference highlights that utilization is more unevenly distributed across wealth groups than insurance enrollment itself.

**Table 2.** Equity in insurance coverage and healthcare use (Erreygers indices).

Outcome	Erreygers E	SE	*p*-value
Insurance coverage	+0.0255***	0.0019	<0.001
Any healthcare use (12 m)	+0.0657***	0.0079	<0.001

Notes: Positive E indicates pro-rich concentration. ***p < 0.001.

### Determinants of healthcare utilization

Table 3 presents survey-weighted logistic regression results. Insurance coverage is strongly associated with healthcare utilization. Insured households have an odds ratio (OR) of 2.19 (95% CI: 1.92–2.50, *p* < 0.001), corresponding to an average marginal effect of +14.6% points. Female-headed households also show higher utilization (OR = 1.31, 95% CI: 1.22–1.42, *p* < 0.001). Urban residence does not differ significantly from rural households (OR = 0.99, 95% CI: 0.90–1.10, *p* = 0.873). Wealth gradients are evident. Compared to the poorest quintile, the richest quintile has 50% higher odds of utilization (OR = 1.50, 95% CI: 1.32–1.70, *p* < 0.001). The middle quintile shows OR = 1.22 (95% CI: 1.08–1.39, *p* = 0.002), and the second quintile is not statistically different from the poorest (OR = 1.10, 95% CI: 0.96–1.26, *p* = 0.152).

**Table 3.** Determinants of healthcare use (N **=** 15,039).

Variable	Coef.	SE	OR	95% CI (OR)	*p*-value
**Health Insurance Coverage**					
Uninsured	Ref				
Insured	0.784***	0.066	2.19	(1.92, 2.50)	<0.001
**Household Headed**					
Male-headed	Ref				
Female-headed	0.273***	0.039	1.31	(1.22, 1.42)	<0.001
**Residence**					
Rural Residence	Ref				
Urban residence	−0.008	0.051	0.99	(0.90, 1.10)	0.873
**Wealth Category**					
Wealth Q1, Poorest	Ref				
Wealth Q2	0.097	0.068	1.10	(0.96, 1.26)	0.152
Wealth Q3	0.202**	0.066	1.22	(1.08, 1.39)	0.002
Wealth Q4	0.326***	0.064	1.39	(1.22, 1.57)	<0.001
Wealth Q5 (richest)	0.406***	0.064	1.50	(1.32, 1.70)	<0.001
**VUP Participation**					
No Participation	Ref				
Direct Support	0.393***	0.066	1.48	(1.30, 1.69)	<0.001
Classic PW	0.247***	0.062	1.28	(1.13, 1.45)	<0.001
Expanded PW	0.352***	0.064	1.42	(1.25, 1.61)	<0.001
NSDS	0.304***	0.066	1.36	(1.19, 1.54)	<0.001

Notes: Reference categories: Rural residence; Wealth quintile 1 (poorest); No participation in VUP components; Uninsured. Estimates survey-weighted with district fixed effects. OR = odds ratio; CI = confidence interval. Average marginal effect of insurance: +14.6% points (95% CI: 12.8–16.4). ***p < 0.001, **p < 0.01, *p < 0.05.

Table 3 shows also that participation in VUP components is consistently associated with higher utilization. Direct Support shows OR = 1.48 (95% CI: 1.30–1.69, *p* < 0.001). Classic Public Works shows OR = 1.28 (95% CI: 1.13–1.45, *p* < 0.001). Expanded Public Works shows OR = 1.42 (95% CI: 1.25–1.61, *p* < 0.001). Nutrition-Sensitive Direct Support shows OR = 1.36 (95% CI: 1.19–1.54, *p* < 0.001). Overall, the strongest associations with healthcare utilization are observed for insurance coverage and Direct Support participation, both with odds ratios above 1.4 and narrow confidence intervals.

### Causal effects of insurance

Table 4 presents causal estimates of insurance on healthcare utilization. The kernel-based propensity score matching (PSM) estimator shows that insurance increases utilization by 12.5% points (SE = 0.91, *p* < 0.001) with 95% confidence interval is approximately 10.7 to 14.3% points. The inverse-probability-weighted regression adjustment (IPWRA) yields a nearly identical estimate of 12.3% points (SE = 0.94, *p* < 0.001), with a 95% confidence interval of 10.5 to 14.1% points.

**Table 4.** Estimated effects of insurance on healthcare utilization.

Method	Effect (pp)	SE	*p*-value
PSM – Nearest neighbor (ATT)	6.9	5.04	0.170
PSM – Kernel matching (ATT)	12.5***	0.91	<0.001
IPWRA (ATE)	12.3***	0.94	<0.001

Notes: ATT = Average Treatment Effect on the Treated; ATE = Average Treatment Effect. Survey weights applied. ***p < 0.001.

In contrast, the nearest-neighbor PSM estimate shows lower precision (SE = 5.04, 95% CI: −2.9 to 16.7% points) compared to kernel PSM and IPWRA. This reflects inherent trade-offs in matching estimators: nearest-neighbor matching uses fewer observations for each comparison and is more sensitive to matching quality, particularly in finite samples with heterogeneous covariate distributions. The wide confidence interval encompasses the point estimates from kernel PSM (12.5 pp) and IPWRA (12.3 pp), suggesting consistency across methods but highlighting the efficiency advantages of kernel weighting and doubly robust estimation. Greater confidence is therefore placed in the kernel PSM and IPWRA results, which leverage more information and provide more stable estimates. Both preferred estimators yield treatment effects of approximately 12% points, representing nearly a 50% increase relative to baseline utilization of 25.5%.

## Discussion

This study examined whether Rwanda’s near-universal health insurance system has translated into equitable healthcare utilization in sectors participating in the Vision Umurenge Programme (VUP). Four results are central. First, insurance coverage was exceptionally high at 86%, however only one in four individuals reported using healthcare in the past year, revealing a persistent gap between formal coverage and actual service use. Second, while coverage showed only mild pro-rich inequality, utilization displayed a considerably stronger pro-rich gradient. Third, regression and causal analyses confirmed that insurance substantially increased the likelihood of healthcare use, with a 12-percentage point gain relative to the uninsured. Finally, participation in VUP components was consistently associated with additional improvements in utilization, particularly among the poorest households.

The particularly strong association between Direct Support and healthcare utilization (OR = 1.48, 95% CI: 1.30–1.69) relative to other VUP components warrants mechanism-focused interpretation. Direct Support provides unconditional cash transfers without labor requirements, offering immediate liquidity that households can deploy flexibly to cover transport costs, opportunity costs of time, and other indirect expenses associated with care-seeking. In contrast, Public Works components while valuable for poverty reduction and livelihood support require substantial time commitments that may compete with healthcare access, particularly for acute illness episodes requiring same-day travel to facilities.

The conditionality-free structure of Direct Support aligns with broader evidence from cash transfer programs showing that unconditional transfers are particularly effective at reducing barriers to healthcare when illness timing is unpredictable and immediate liquidity is critical for transport, meals, and foregone income. This mechanism likely explains why Direct Support shows the strongest utilization effect despite all VUP components targeting the same vulnerable populations and providing similar overall resource transfers. The finding reinforces arguments for unconditional social protection as a complement to health insurance in settings where indirect costs constitute major access barriers [10,13].

These results were consistent with previous studies demonstrating that health insurance raises healthcare use but does not guarantee equity. Prior analyses in Rwanda reported that insured individuals were between two and nearly five times more likely to use services than the uninsured [20,21], closely aligned with the present estimate that insurance more than doubled the odds of care-seeking (OR: 2.19). Similarly, the finding that only 25% of insured individuals reported healthcare use supports earlier evidence showing utilization of 20–25% despite coverage rates above 86–90% [20,22]. These findings also align with prior work that insurance provides strong financial protection, reducing catastrophic health expenditure by up to four-fold [21], but with persistent inequalities in access.

At the same time, the results contradict the expectation that Rwanda’s subsidy-based model has fully equalized access. We found that utilization was nearly three times more unequal than coverage (Erreygers index +0.066 vs. +0.026), echoing broader concerns about inequity in health systems. This contrasts with earlier claims that Rwanda’s financing reforms had delivered fully pro-poor outcomes [2]. Instead, the results are closer to findings from other sub-Saharan African contexts. In Ethiopia, insured non-poor households were twice as likely to use services as the insured poor [23]. In Ghana, NHIS enrollees from wealthier quintiles had 1.8 times higher odds of outpatient visits [17]. In Kenya, NHIF members in the richest quintile had utilization rates nearly double those of the poorest [11]. Together, this evidence underscores that while subsidies remove direct payment barriers, they do not address indirect costs such as transport or lost income, which disproportionately affect the poorest households.

Findings on service-specific utilization suggest further nuance. Previous research showed that CBHI particularly benefits married women and children under five [3] and that maternal health services displayed consistent pro-poor effects [17]. By contrast, the broader measure of general healthcare utilization reveals persistent inequities [24]. This divergence may reflect differences in outcome measures: insurance appears more effective in promoting equity for targeted services, such as maternal care [25], than in ensuring equitable use across the full spectrum of healthcare needs [26].

The added value of VUP emerges clearly in this analysis. Households participating in Direct Support had 48% higher odds of utilization, those in Classic Public Works had 28% higher odds, Expanded Public Works increased odds by 42%, and Nutrition-Sensitive transfers by 36%. These sizeable effects confirm that VUP systematically enhances healthcare use. This supports evidence from other settings that social protection reduces liquidity constraints and opportunity costs, enabling households to make fuller use of insured services [12]. The programme’s explicit focus on the poorest households is central to its value: by offsetting transport costs, compensating for lost income, and addressing nutritional vulnerabilities, VUP addresses barriers that insurance alone cannot resolve. In doing so, it strengthens the translation of formal coverage into effective and equitable use of services.

This study has several important limitations and must be acknowledged. This study has several important limitations. First, the cross-sectional design precludes assessment of dynamic effects and temporal pathways. We cannot determine whether sustained VUP participation leads to cumulative improvements in healthcare access over time, nor can we rule out reverse causality where healthier households are more likely to maintain employment in public works programs. Longitudinal data would enable analysis of how social protection influences health-seeking trajectories and whether insurance-utilization relationships strengthen or weaken with duration of coverage.

Second, healthcare utilization is self-reported with 12-month recall, potentially subject to recall bias and social desirability bias. Recall accuracy may also vary systematically by socioeconomic status, as wealthier and more educated households may be more likely to recall and report routine healthcare episodes; this differential recall could inflate the observed pro-rich concentration in utilization. While EICV7 employs validated survey instruments and trained enumerators, individuals may underreport minor illnesses deemed insignificant or overestimate care-seeking to provide socially desirable responses. Administrative data linkage with facility records could validate self-reported utilization patterns in future research. Third, despite robust covariate adjustment and multiple causal estimators, residual confounding from unobserved factors remains possible. Variables such as health beliefs, social networks, trust in the health system, and past healthcare experiences may influence both insurance enrollment and utilization but are not measured in EICV7. Instrumental variable approaches leveraging exogenous policy variation or quasi-experimental designs could strengthen causal identification.

Fourth, restricting the analysis to VUP sectors enhances policy relevance by focusing on the most vulnerable populations but limits generalizability to non-VUP areas. VUP sectors are systematically poorer and more rural than national averages, potentially showing different insurance-utilization relationships. Future comparative work could examine whether VUP and non-VUP sectors exhibit differential treatment effects and explore mechanisms underlying any observed heterogeneity. Finally, EICV7 does not measure healthcare quality, appropriateness of care, or health outcomes. Increased utilization may not translate to improved health if services are clinically ineffective, culturally inappropriate, or poorly targeted to actual health needs. Supply-side facility assessments and health outcome evaluations would complement this demand-side analysis and provide a more complete picture of effective coverage.

## Conclusion

Rwanda has achieved near-universal health insurance coverage, yet only a quarter of insured individuals reported using healthcare in the past year, revealing a persistent gap between financial entitlement and effective access. Insurance substantially increases the likelihood of care-seeking, but utilization remains pro-rich, with inequities nearly three times greater than those observed for coverage. These results confirm that financial protection through insurance, while necessary, is insufficient to achieve equitable healthcare use.

The added value of the Vision Umurenge Programme (VUP) lies in its explicit focus on the poorest households, who face indirect costs, foregone income, and nutritional vulnerabilities that insurance alone cannot address. By reducing these barriers, VUP complements insurance and expands effective coverage. This layered approach illustrates how integrating social protection with health financing can close persistent equity gaps. In moving toward universal health coverage, Rwanda’s experience highlights a broader lesson for low- and middle-income countries: expanding insurance is essential but must be paired with targeted social protection to ensure that the most vulnerable are not left behind.

Future research should extend this analytical framework to other low- and middle-income countries with expanding insurance coverage and complementary social protection programs. Cross-country comparative studies could identify generalizable mechanisms linking social protection to healthcare equity and examine how context-specific factors such as health system capacity, provider payment incentives, cultural norms around care-seeking, and program design features moderate these relationships. Comparative evidence across diverse institutional settings would strengthen the empirical foundation for integrated health financing and social protection strategies globally.

## Data Availability

The data that support the findings of this study are available from the National Institute of Statistics of Rwanda (NISR), subject to a formal data request and approval process. The dataset is publicly cataloged as: RWA-NISR-EICV7-2023–2024-v01: Seventh Integrated Household Living Conditions Survey (EICV7) dataset for public distribution. Data were collected between October 2023 and October 2024 across nine survey cycles to capture seasonal variation in household consumption and living conditions. Access to the dataset can be requested through the NISR Microdata Catalog: https://microdata.statistics.gov.rw/index.php/catalog Restrictions apply to the availability of these data, which were used under license for the current study and are not publicly downloadable without authorization from NISR.

## References

[1] Dupas P, Jain R. Women left behind: gender disparities in utilization of government health insurance in India. Am Econ Rev. 2024;114:3345–10. doi: 10.1257/aer.20230521

[2] Sayinzoga F, Bijlmakers L. Drivers of improved health sector performance in Rwanda: a qualitative view from within. BMC Health Serv Res. 2016;16:123. doi: 10.1186/s12913-016-1351-4 27059319 PMC4826525

[3] Woldemichael A, Gurara D, Shimeles A. The impact of community based health insurance schemes on out-of-pocket healthcare spending: evidence from Rwanda. IMF Work Pap. 2019;19:1. doi: 10.5089/9781484398074.001

[4] Lu C, Chin B, Lewandowski JL, et al. Towards universal health coverage: an evaluation of Rwanda Mutuelles in its first eight years. PLoS One. 2012;7:e39282. doi: 10.1371/journal.pone.0039282 22723985 PMC3377670

[5] Lu C, Mejia-Guevara I, Hill K, et al. Community-based health financing and child stunting in rural Rwanda. Am J Public Health. 2016;106:49–55. doi: 10.2105/AJPH.2015.302913 26562109 PMC4743535

[6] Chirwa GC, Suhrcke M, Moreno-Serra R. Socioeconomic inequality in premiums for a community-based health insurance scheme in Rwanda. Health Policy Plan. 2021;36:14–25. doi: 10.1093/heapol/czaa135 33263730

[7] Nisr R. Seventh Integrated Household Living Conditions Survey (EICV7) report. Kigali, Rwanda: National Institute of Statistics of Rwanda; 2025.

[8] Koch R, Nkurunziza T, Rudolfson N, et al. Does community-based health insurance protect women from financial catastrophe after cesarean section? A prospective study from a rural hospital in Rwanda. BMC Health Serv Res. 2022;22:717. doi: 10.1186/s12913-022-08101-3 35642031 PMC9153099

[9] Liu K, Subramanian SV, Lu C. Assessing national and subnational inequalities in medical care utilization and financial risk protection in Rwanda. Int J Equity Health. 2019;18:51. doi: 10.1186/s12939-019-0953-y 30917822 PMC6437855

[10] Muremyi R, Migisha LB, Pasteur ML, et al. Barriers to health insurance uptake in Rwanda: a nationwide cross-sectional survey. Pan Afr Med J. 2025;51:51. doi: 10.11604/pamj.2025.51.8.45920 40727520 PMC12296669

[11] Barasa E, Kazungu J, Nguhiu P, et al. Examining the level and inequality in health insurance coverage in 36 Sub-Saharan African countries. BMJ Glob Health. 2021;6:e004712. doi: 10.1136/bmjgh-2020-004712 PMC8076950 33903176

[12] Van Hees SGM, O’Fallon T, Hofker M, et al. Leaving no one behind? Social inclusion of health insurance in low- and middle-income countries: a systematic review. Int J Equity Health. 2019;18:134. doi: 10.1186/s12939-019-1040-0 31462303 PMC6714392

[13] Mori AT, Mudenda M, Robberstad B, et al. Impact of cash transfer programs on healthcare utilization and catastrophic health expenditures in rural Zambia: a cluster randomized controlled trial. Front Health Serv. 2024;4:1254195. doi: 10.3389/frhs.2024.1254195 38741917 PMC11089190

[14] Ngamasana EL, Moxie J. Cash transfer, maternal and child health outcomes: a scoping review in sub-Saharan Africa. Glob Health Action. 2024;17:2309726. doi: 10.1080/16549716.2024.2309726 38333923 PMC10860414

[15] Anjorin SS, Ayorinde AA, Abba MS, et al. Equity of national publicly funded health insurance schemes under the universal health coverage agenda: a systematic review of studies conducted in Africa. J Public Health. 2022;44:900–909. doi: 10.1093/pubmed/fdab316 34390345

[16] Fenny AP, Yates R, Thompson R. Strategies for financing social health insurance schemes for providing universal health care: a comparative analysis of five countries. Glob Health Action. 2021;14:1868054. doi: 10.1080/16549716.2020.1868054 33472557 PMC7833020

[17] Wang W, Temsah G, Mallick L. The impact of health insurance on maternal health care utilization: evidence from Ghana, Indonesia and Rwanda. Health Policy Plan. 2016:czw135. doi: 10.1093/heapol/czw135 PMC5400062 28365754

[18] Eze P, Ilechukwu S, Lawani LO. Impact of community-based health insurance in low- and middle-income countries: a systematic review and meta-analysis. PLoS One. 2023;18:e0287600. doi: 10.1371/journal.pone.0287600 37368882 PMC10298805

[19] Wagstaff A, van Doorslaer E. Income inequality and health: what does the literature tell us?. Annu Rev Public Health. 2000;21:543–567. doi: 10.1146/annurev.publhealth.21.1.543. Cited in: PMID: 10884964. 10884964

[20] Muremyi R, Dominique H, Ignace K, et al. Prediction of out-of-pocket health expenditures in Rwanda using machine learning techniques. Pan Afr Med J. 2020;37:37. doi: 10.11604/pamj.2020.37.357.27287 PMC7992429 33796171

[21] Saksena P, Antunes AF, Xu K, et al. Mutual health insurance in Rwanda: evidence on access to care and financial risk protection. Health Policy. 2011;99:203–209. doi: 10.1016/j.healthpol.2010.09.009 20965602

[22] Muremyi R, Haughton D, Niragire F, et al. A cross-sectional analysis of the impact of health insurance on the use of health care in Rwanda. Heliyon. 2023;9:e17086. doi: 10.1016/j.heliyon.2023.e17086 37484315 PMC10361221

[23] Shimeles A. Community based health insurance schemes in Africa: the case of Rwanda. 2010.

[24] Yokobori Y, Kiyohara H, Mulati N, et al. Roles of social protection to promote health service coverage among vulnerable people toward achieving universal health coverage: a literature review of international organizations. Int J Environ Res Public Health. 2023;20:5754. doi: 10.3390/ijerph20095754 37174271 PMC10177917

[25] Hunter BM, Murray SF. Demand-side financing for maternal and newborn health: what do we know about factors that affect implementation of cash transfers and voucher programmes? BMC Pregnancy Childbirth. 2017;17:262. doi: 10.1186/s12884-017-1445-y 28854877 PMC5577737

[26] Doshmangir L, Bazyar M, Rashidian A, et al. Iran health insurance system in transition: equity concerns and steps to achieve universal health coverage. Int J Equity Health. 2021;20:37. doi: 10.1186/s12939-020-01372-4 33446202 PMC7807408

